# Effects of rifampin, itraconazole and esomeprazole on the pharmacokinetics of alisertib, an investigational aurora a kinase inhibitor in patients with advanced malignancies

**DOI:** 10.1007/s10637-017-0499-z

**Published:** 2017-08-30

**Authors:** Xiaofei Zhou, Shubham Pant, John Nemunaitis, A. Craig Lockhart, Gerald Falchook, Todd M. Bauer, Manish Patel, John Sarantopoulos, Michael Bargfrede, Andreas Muehler, Lakshmi Rangachari, Bin Zhang, Karthik Venkatakrishnan

**Affiliations:** 1 Millennium Pharmaceuticals, Inc., a wholly owned subsidiary of Takeda Pharmaceutical Company Limited, Cambridge, MA USA; 2 Oklahoma University Medical Center, Oklahoma City, OK USA; 3 Mary Crowley Cancer Research Centers, Dallas, TX USA; 4 Washington University, St. Louis, MO USA; 5 Sarah Cannon Research Institute at HealthONE, Denver, CO USA; 6 Sarah Cannon Research Institute, Nashville, TN USA; 7 Florida Cancer Specialists, Sarasota, FL USA; 8 Institute for Drug Development, Cancer Therapy and Research Center, University of Texas Health Science Center San Antonio, San Antonio, TX USA; 9 MJB Pharma Consulting, Inc, Cambridge, MA USA; 10 Seqirus Pharmaceuticals, Cambridge, MA USA

**Keywords:** Alisertib, Drug-drug interactions, CYP3A4, Inhibition, Induction, Aurora A kinase

## Abstract

*Aim* Two studies investigated the effect of gastric acid reducing agents and strong inducers/inhibitors of CYP3A4 on the pharmacokinetics of alisertib, an investigational Aurora A kinase inhibitor, in patients with advanced malignancies. *Methods* In Study 1, patients received single doses of alisertib (50 mg) in the presence and absence of either esomeprazole (40 mg once daily [QD]) or rifampin (600 mg QD). In Study 2, patients received single doses of alisertib (30 mg) in the presence and absence of itraconazole (200 mg QD). Blood samples for alisertib and 2 major metabolites were collected up to 72 h (Study 1) and 96 h (Study 2) postdose. Area under the curve from time zero extrapolated to infinity (AUC_0-inf_) and maximum concentrations (C_max_) were calculated and compared using analysis of variance to estimate least squares (LS) mean ratios and 90% confidence intervals (CIs). *Results* The LS mean ratios (90% CIs) for alisertib AUC_0-inf_ and C_max_ in the presence compared to the absence of esomeprazole were 1.28 (1.07, 1.53) and 1.14 (0.97, 1.35), respectively. The LS mean ratios (90% CIs) for alisertib AUC_0-inf_ and C_max_ in the presence compared to the absence of rifampin were 0.53 (0.41, 0.70) and 1.03 (0.84, 1.26), respectively. The LS mean ratios (90% CIs) for alisertib AUC_0-inf_ and C_max_ in the presence compared to the absence of itraconazole were 1.39 (0.99, 1.95) and 0.98 (0.82, 1.19), respectively. *Conclusions* The use of gastric acid reducing agents, strong CYP3A inhibitors or strong metabolic enzyme inducers should be avoided in patients receiving alisertib.

## Introduction

Alisertib is a selective small molecule inhibitor of Aurora A kinase that is being developed for the treatment of advanced malignancies. The recommended dose of alisertib for clinical development as a single agent in Western patient populations is 50 mg BID administered for 7 days in 21-day cycles [1–3]. This dose and schedule has been used in multiple Phase 2 clinical studies in solid tumor and hematologic malignancies, with clinical antitumor activity observed in small cell lung cancer, breast cancer, head and neck cancer, gastroesophageal adenocarcinoma, ovarian cancer, and various hematologic malignancies [4–7]. Alisertib has also demonstrated antitumor activity in Phase 2 studies in ovarian cancer [7] and small cell lung cancer [8] at a dose of 40 mg BID administered on Days 1–3, 8–10, 15–17 in combination with weekly paclitaxel (60 mg/m^2^ on Days 1, 8 and 15) in 28-day cycles. Identification of further appropriate combination partners and sensitive patient populations is anticipated to ensure that an acceptable risk/benefit profile can be achieved. Aurora A has been implicated in the development of resistance to multiple chemotherapies and targeted agents and preclinical data suggest that alisertib can be combined with multiple therapies to yield additive or synergistic antitumor activity. Furthermore, combinations with targeted therapies might yield more favorable clinical risk/benefit profiles than combinations with chemotherapeutic partners due to decreased risk for overlapping toxicities [9]. Most common treatment related toxicities observed with alisertib were fatigue and toxicities related to the antiproliferative mechanism of action, namely neutropenia and stomatitis.

Alisertib has been shown to be extensively metabolized in humans [10] by both oxidation and glucuronidation pathways. Direct acyl glucuronidation results in the formation of metabolite, M1 whereas CYP-mediated *O-*demethylation of the fluoromethoxyphenyl moiety results in the formation of metabolite M2, representing the two primary metabolites of alisertib. Studies in human liver microsomes suggest the involvement of multiple cytochrome P450 (CYP) isozymes and uridine diphosphate-glucuronosyltransferase (UGT) isozymes. Multiple UGT enzymes (UGT1A1, 1A3, and 1A8) were involved in alisertib metabolism based on in vitro data. Genotyping of UGT1A1 was performed in more than 300 patients. The impact of UGT1A1 genotype was not identified as a significant covariate on the apparent oral clearance of alisertib [3]. CYP3A4 was the major CYP isozyme contributing to the oxidative metabolism of alisertib and it is estimated that CYP3A4-mediated metabolism may account for approximately 60% of alisertib total clearance (Takeda data on file). Given the importance of CYP3A4 and glucuronidation to alisertib clearance, moderate and strong inhibitors of CYP3A4, and clinically significant inducers of CYP3A4/UGT enzymes have the potential to alter the systemic exposure of alisertib. The contribution of CYP3A4 to alisertib biotransformation also exceeds the 25% threshold of potential clinical relevance for drug-drug interactions (DDIs) based on the US Food and Drug Administration (FDA) and European Medicines Agency (EMA) guidance documents [11, 12].

Alisertib is an acidic drug (pKa of 4.53 for the free acid) with low aqueous solubility at acidic pH. To bypass the stomach and delay dissolution until delivery to the upper small intestine, alisertib is formulated as an enteric-coated tablet (ECT). Proton pump inhibitors (PPIs) inhibit gastric acid secretion, and therefore have the potential to interfere with the absorption of drugs/dosage forms for which gastric pH is an important determinant of dissolution, absorption and bioavailability [13]. The use of gastric acid reducing agents is particularly prevalent in cancer patient populations [14], making the assessment of potential DDIs especially important in the development of orally administered anticancer agents with pH-dependent solubility [15].

To investigate the potential for clinically relevant DDIs, two clinical studies were conducted. Study 1 investigated the effects of rifampin, a strong metabolic inducer of pregnane X receptor (PXR)-inducible drug-metabolizing enzymes (eg, CYP3A), and esomeprazole, a PPI on the pharmacokinetics of alisertib ECTs. Study 2 investigated the effect of itraconazole, a strong CYP3A4 inhibitor, on the pharmacokinetics of alisertib ECTs. Alisertib is a cytotoxic agent and as it cannot be administered to healthy subjects, these studies were conducted in patients with advanced solid tumors or lymphomas. Given the high likelihood of polypharmacy in advanced cancer patients, these studies were important to assess the potential risk for clinically meaningful alterations in alisertib exposure with co-administered drugs and to guide DDI risk management in the clinical development program [16].

## Methods

### Study design

**Fig. 1:**
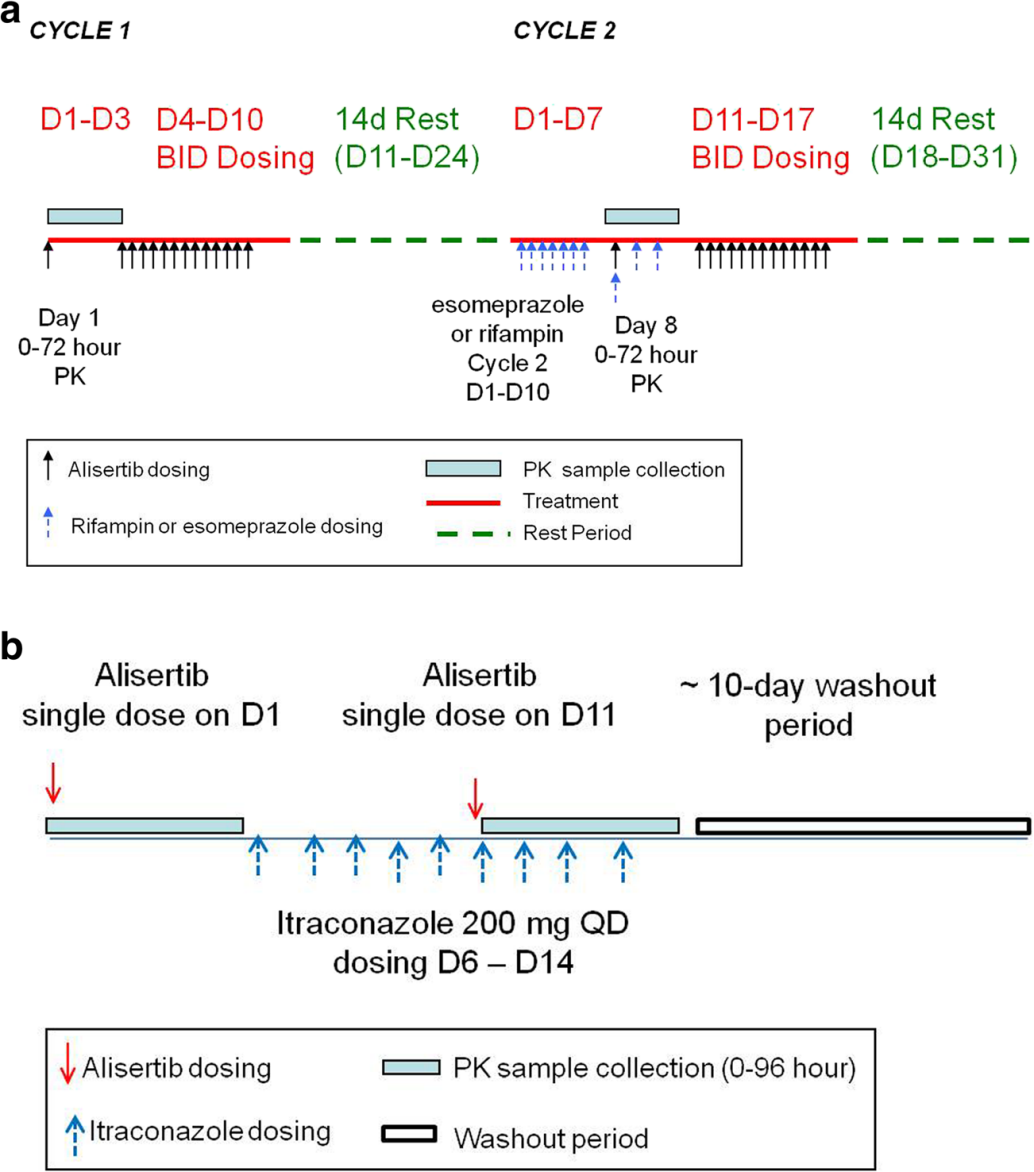
Study schema for assessment of effects of esomeprazole, rifampin (**a**) and itraconazole (**b**) on pharmacokinetics of alisertib. D: day; BID, twice daily; PK, pharmacokinetics

Study 1 was a phase 1, open-label, fixed sequence, 2-cycle study in patients with advanced solid tumors or lymphomas. A primary objective of the study was to evaluate the effect of multiple doses of esomeprazole and rifampin on the pharmacokinetics of a single 50-mg dose of alisertib administered as ECTs. Another objective was to evaluate the effect of single and multiple doses of alisertib on the QTc interval. The methods and results of the QT analysis will be reported separately, and hence only details pertinent to the DDI assessment are provided here (Fig. 1, panel A).

Patients were screened up to 28 days prior to the first dose of alisertib to assess eligibility. Eligible patients were then enrolled into the study and received a single dose of 50 mg alisertib on Day 1 of Cycle 1 and Day 8 of Cycle 2. In Cycle 2, patients also received single daily doses of either esomeprazole (40 mg QD delayed-release capsules) or rifampin (600 mg once daily [QD]) on Days 1 through 10. On Day 8, the dose of alisertib was either administered 1 h after esomeprazole or co-administered with rifampin (as appropriate). Patients also received 50 mg BID alisertib in Cycle 1 (Day 4 through until the morning dose on Day 10) and Cycle 2 (Day 11 through until the morning of Day 17). Day 1 of Cycle 2 started after a 2-week washout following alisertib dosing in Cycle 1. Beginning Cycle 3, patients received alisertib 50 mg BID on Days 1–7 in 21-day cycles until disease progression or unacceptable toxicity.

Patients attended the study center on the day prior to the first dose (Day −1) for baseline assessments and returned on each of Days 1 to 4 of Cycle 1 and Days 8 to 11 of Cycle 2 for study assessments. Alisertib was administered in the study center on Day 1 of Cycle 1 and on Day 8 of Cycle 2. On both alisertib dosing days, patients were nil-by-mouth (no food or drink except water) from 2 h prior to alisertib dosing, until completion of the 4-h assessments.

Study 2 was a phase 1, open-label study in patients with advanced solid tumors or relapsed/refractory lymphomas. The primary objective was to assess the effect of multiple dose administration of itraconazole (200 mg QD) on the single-dose pharmacokinetics of alisertib (30 mg) (Fig. 1, panel B).

Patients were screened up to 28 days prior to the first dose of alisertib to assess eligibility. Eligible patients were then enrolled into the study and received single doses of 30 mg alisertib on Day 1 and on Day 10, which were both administered in the study center. Patients also received oral solution doses of 200 mg itraconazole QD on Days 5 through 13. On Day 10, alisertib was administered 1 h after itraconazole. Itraconazole was administered in the study center on Days 5, and 10 through 13 with the remaining doses being administered at home by the patients. All doses of alisertib and itraconazole were taken on an empty stomach with patients being nil-by-mouth (except water) for 2 h before and until 1 h after each dose. The itraconazole dosing regimen and design employed in this study were consistent with current best practice recommendations for the conduct of itraconazole DDI studies in the development of CYP3A4 substrates [17].

Both studies were conducted in accordance with the International Conference on Harmonization guideline for Good Clinical Practice and the ethical principles of the Declaration of Helsinki. Both studies were conducted in the United States (Study 1 involved 6 centers, and Study 2 involved 4 centers) and were approved by the institutional review board(s) and/or local independent ethics committee(s) at each center. The studies were both registered on ClinicalTrials.gov (Study 1: NCT01844583, Study 2: NCT02259010). Study 1 was conducted between June 2013 and August 2014, and Study 2 was conducted between October 2014 and March 2015.

### Patients

Eligible patients were male or female with histologically or cytologically confirmed metastatic and/or advanced solid tumours or lymphomas for which standard curative or life-prolonging treatment did not exist or was no longer effective or tolerable. Patients were aged 18 years or older, had an Eastern Cooperative Oncology Group (ECOG) performance status of 0 or 1. All patients had to provide written informed consent and comply with contraceptive requirements. Key exclusion criteria included treatment with any anticancer therapy or investigational agent within 3 or 4 weeks (or 5 half-lives) prior to Day 1, recurrent nausea and/or vomiting or any known gastrointestinal abnormality or procedures that could interfere with or modify absorption or tolerance to alisertib. Patients were excluded if they had known hypersensitivity to any of the protocol required medications or had any contraindications to those medications. Patients were excluded from both studies if they required treatment with clinically significant enzyme inducers within 14 days prior to Day 1 or during the study and patients with a medical condition requiring use of pancreatic enzymes, or daily, chronic or regular use of proton pump inhibitors or histamine (H2) receptor antagonists. Study 2 also excluded patients taking moderate or strong CYP3A4 inhibitors within 14 days prior to Day 1.

Patient evaluability for assessment of DDI required completion of all protocol-specified PK sampling and dosing for both alisertib and interacting drugs.

### Pharmacokinetic assessments

Blood samples for analysis of alisertib and its metabolites M1 (alisertib acylglurcuronide) and M2 (*O*-desmethyl alisertib) were collected at intervals from 0 (predose) to 72 h after the single doses of alisertib on Day 1 of Cycle 1 and Day 8 of Cycle 2, in Study 1, and at intervals from 0 (predose) to 96 h after the single doses of alisertib on Days 1 and 10 in Study 2.

Plasma samples were analyzed for alisertib and its metabolite concentrations using previously published validated liquid chromatography tandem mass spectrometry (LC-MS/MS) methods utilizing solid phase extraction procedure. [1, 2] In Study 1, the quantitation range for the alisertib assay was 5 to 2500 ng/mL, the assay precision, expressed as percent coefficients of variation (%CV) for quality control (QC) samples ranged from 4.0 to 7.1% and the mean accuracy, expressed as percent bias, for QC samples ranged from −0.8 to 2.0%. The quantitation range for the M1 assay was 2.00 to 1000 ng/mL, the assay precision ranged from 3.0 to 4.1% and the mean accuracy ranged from −1.5 to 0.3%. The quantitation range for the M2 assay was 2.00 to 1000 ng/mL, the assay precision ranged from 3.0 to 4.3% and the mean accuracy ranged from −1.1 to 0.7%. The same assays were used in Study 2, with similar values for assay precision and accuracy.

Pharmacokinetic parameters were estimated where data permitted using non-compartmental analysis with Phoenix™ WinNonlin® Version 6.3 (Pharsight Corporation, Mountain View, CA, USA). The following pharmacokinetic parameters were calculated following each single dose of alisertib in both studies where data permitted: maximum observed plasma concentration (C_max_), and first time to C_max_ (T_max_), area under the concentration time curve from time 0 to the last quantifiable time point (AUC_0-last_), area under the concentration-time curve from time 0 extrapolated to infinite time (AUC_0-inf_), and terminal half-life (t_1/2_). The AUC parameters were estimated using the linear-log trapezoidal rule.

### Safety assessments

Safety was evaluated based on the incidence of adverse events, and changes from baseline in vital signs, ECGs, weight and clinical laboratory results and ECOG status.

### Statistical analysis

The ratios of geometric mean AUC_0-last_, AUC_0-inf_ and C_max_ of alisertib in the presence of interactant (ie, esomeprazole, rifampin or itraconazole) versus alisertib alone (as reference), and associated 2-sided 90% confidence intervals (CIs) were calculated based on within-patient variance calculated via analysis of variance (ANOVA). The analyses utilized a fixed-sequence repeated measures mixed-effects linear model. All inferences were based on least squares (LS) means estimated from this model. All statistical analyses were conducted using SAS version 9.2 (SAS Institute, NC, USA). The sample size for both studies was based on the expected 90% CIs for the ratio of geometric mean AUCs. For Study 1, the within-patient CV was estimated to be 39.4% based on prior clinical PK data in cancer patients. Assuming the alisertib AUC ratio in the presence versus absence of each interactant was X, a sample size of 18 patients per arm was expected to provide 90% CIs of 0.8X to 1.25X. For Study 2, the within-patient CV was estimated to be 31% based on more recent clinical PK data and a sample size of 16 patients was expected to provide 90% CIs of 0.83X, 1.21X.

## Results

### Patient disposition and demographics

In Study 1, a total of 55 patients were enrolled and 38 (69%) patients completed the protocol specified dosing and pharmacokinetic sampling requirements for DDI assessments in Cycles 1 and 2. Of these 38 patients, 18 patients were in the esomeprazole arm and 20 patients were in the rifampin arm. Overall, the median age of the patients was 61 years (range 32 to 80 years) and the majority of patients were women (64%), White (87%) and not Hispanic or Latino (84%). Fifty four patients had advanced solid tumors and one patient had mantle cell lymphoma.

In Study 2, a total of 24 patients were enrolled and 19 (79%) completed the protocol specified dosing and pharmacokinetic sampling requirements. The median age of the patients was 64 years (range 34 to 76 years), the majority of patients were women (54%), White (83%), and not Hispanic or Latino (83%). All patients had advanced solid tumors.

### Pharmacokinetics

**Fig. 2:**
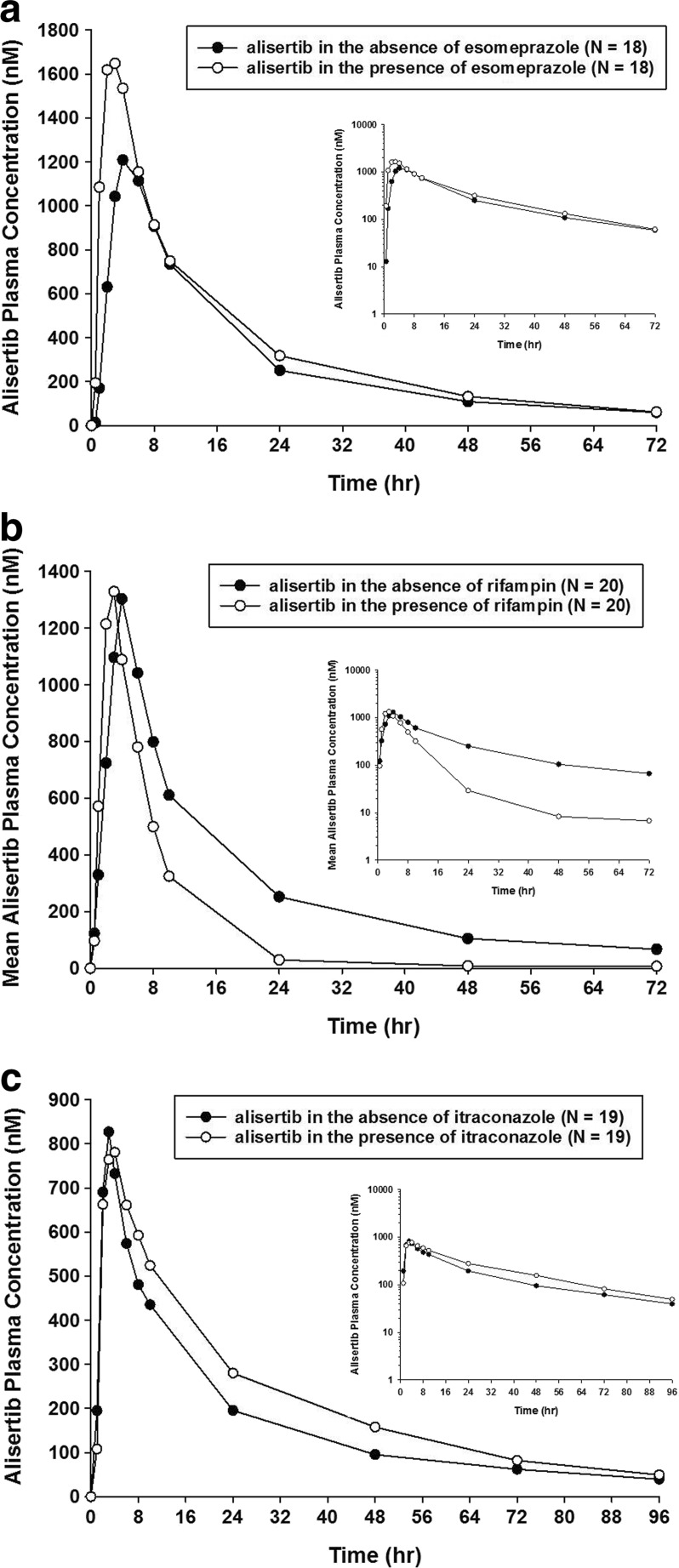
Alisertib plasma concentration-time profiles in the presence and absence of esomeprazole (**a**), rifampin (**b**) and itraconazole (**c**). The insets are alisertib concentration-time profiles in semi-log scales

**Fig. 3:**
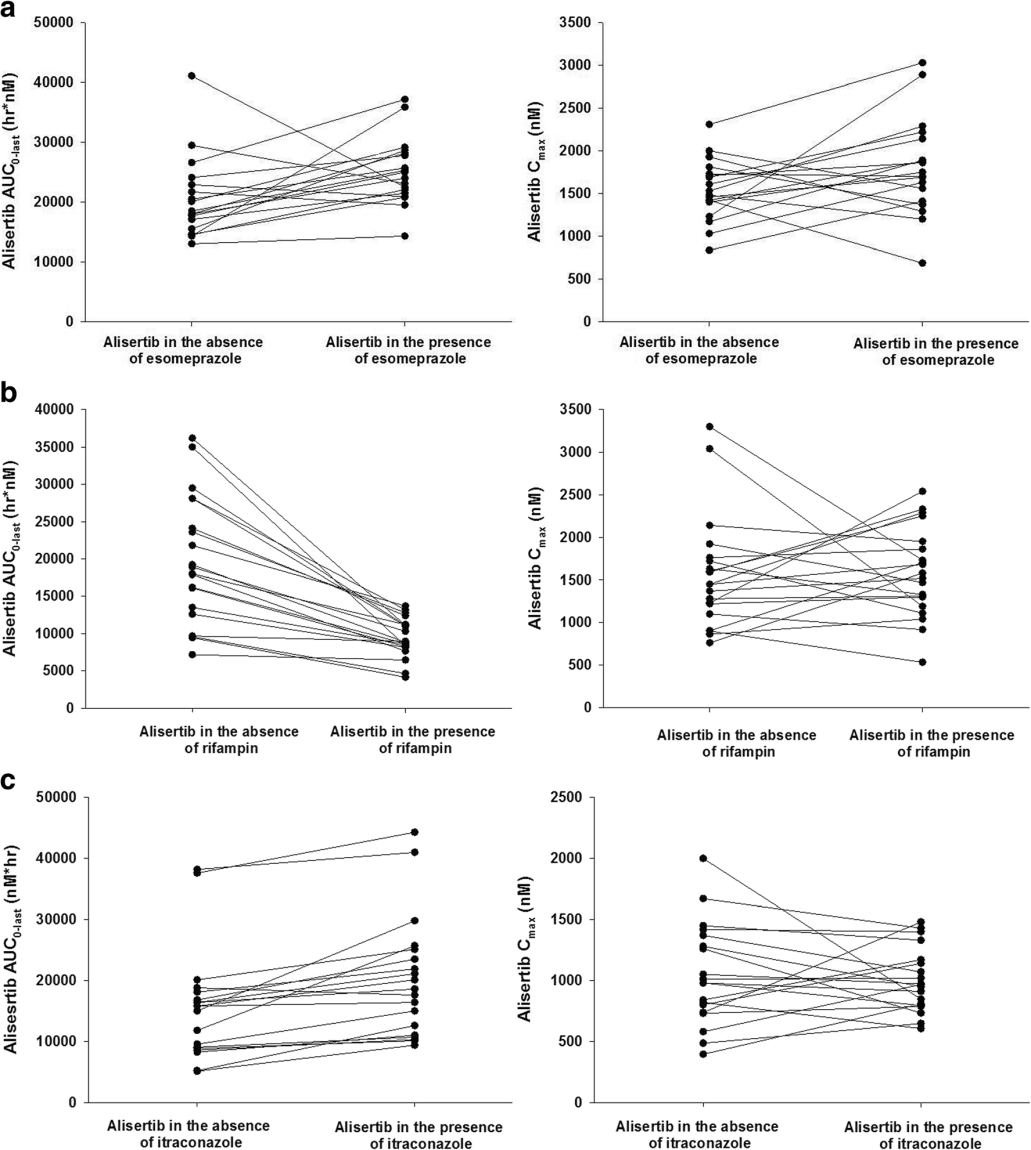
Individual comparisons of alisertib C_max_ and AUC_0-last_ in the presence and absence of esomeprazole (**a**), rifampin (**b**) and itraconazole (**c**)

The mean plasma concentration-time profiles for alisertib in the presence and absence of the interactants are shown in Fig. 2 and the pharmacokinetic parameters for alisertib and its metabolites M1 and M2 are shown in Table 1, along with the results of the statistical analysis of alisertib pharmacokinetic parameters in the presence and absence of the interactants. The individual comparison of alisertib PK parameters in the presence and absence of the interactants are presented in Fig. 3.

**Table 1:** Summary of single dose pharmacokinetic parameters for alisertib and its major metabolites in the presence and absence of interactants

Study/Treatment Arm	Analyte	N	Pharmacokinetic Parameters^**a**^				
			C_max_ (nM)	AUC_0-last_ (nM*h)	AUC_0-inf_ (nM*h)	T_max_ (h)	t_1/2_ (h)
Study 1/ alisertib (50 mg) +/− esomeprazole (40 mg QD)	Alisertib (50 mg)						
	Absence of esomeprazole	18	1501 (23%)	19,563 (33%)	20,251 (38%)^b^	4 (2, 8)	16.1 (5.4)^b^
	Presence of esomeprazole	18	1715 (32%)	24,518 (22%)	25,859 (25%)^c^	3 (1, 8)	16.0 (5.2)^c^
	LS mean ratio (90% CI)	18	1.14 (0.97, 1.35)	1.25 (1.08, 1.45)	1.28 (1.07, 1.53)	-	-
	Alisertib metabolite M1						
	Absence of esomeprazole	18	259 (76%)	4349 (164%)	3443 (77%)^d^	4 (3, 10)	15.2 (4.9)^d^
	Presence of esomeprazole	18	364 (116%)	6944 (182%)	6513 (220%)^e^	3 (1, 24)	17.5 (7.0)^e^
	Alisertib metabolite M2						
	Absence of esomeprazole	18	214 (37%)	8832 (48%)	9512 (21%)^f^	10 (8, 48)	22 (4.2)^f^
	Presence of esomeprazole	18	215 (28%)	9528 (29%)	10,828 (25%)^g^	9.9 (4, 71)	28 (9.2)^g^
Study 1/ alisertib (50 mg) +/− rifampin (600 mg QD)	Alisertib (50 mg)						
	Absence of rifampin	20	1450 (42%)	17,936 (43%)	16,250 (36%)^h^	4 (1, 23)	16.3 (6.6)^h^
	Presence of rifampin	20	1491 (33%)	9062 (28%)	8654 (27%)^i^	2.1 (1, 6)	8.2 (5.5)^i^
	LS mean ratio (90% CI)	20	1.03 (0.84, 1.26)	0.51 (0.41, 0.62)	0.53 (0.41, 0.70)	-	-
	Alisertib metabolite M1						
	Absence of rifampin	20	320 (74%)	5255 (91%)	4709 (124%)^b^	4 (2, 23)	17.8 (7.4)^b^
	Presence of rifampin	20	903 (36%)	8626 (45%)	9361 (48%)^d^	3 (2, 8)	12.0 (8.7)^d^
	Alisertib metabolite M2						
	Absence of rifampin	20	151 (61%)	5586 (65%)	3918 (13%)^j^	10 (4, 24)	19.1 (2.8)^j^
	Presence of rifampin	20	266 (34%)	4589 (44%)	5382 (45%)^b^	6 (3, 10)	16.8 (7.0)^b^
Study 2/alisertib (30 mg) +/− itraconazole (200 mg QD)	Alisertib (30 mg)						
	Absence of itraconazole	19	986 (38%)	13,450 (59%)	14,834 (55%)^k^	2.9 (1.1, 10)	22.6 (10.3)^k^
	Presence of itraconazole	19	971 (26%)	18,215 (49%)	20,558 (57%)^l^	2.9 (1.8, 7.8)	25.4 (9.2)^l^
	LS mean ratio (90% CI)	19	0.98 (0.82, 1.19)	1.35 (1.02, 1.79)	1.39 (0.99, 1.95)	-	-
	Alisertib metabolite M1						
	Absence of itraconazole	18	287 (117%)	4962 (164%)	5758 (174%)^d^	3.1 (1, 23)	23.0 (11.2)^h^
	Presence of itraconazole	18	279 (148%)	6720 (187%)	7087 (198%)^l^	3.8 (2, 10)	26.8 (10.2)^c^
	Alisertib metabolite M2						
	Absence of itraconazole	18	100 (46%)	4977 (58%)	4926 (41%)^i^	9.4 (3.0, 48)	28 (9.2)^i^
	Presence of itraconazole	19	82 (96%)	4390 (66%)	4120 (27%)^j^	23.6 (5.7, 95)	30.6 (5.8)^j^

^a^Values are geometric means (% coefficient of variation) for C_max_ and AUC parameters, median (minimum, maximum) for T_max_, and arithmetic mean (standard deviation) for t_1/2_. b *N* = 14, c *N* = 16, d *N* = 11, e *N* = 17, f *N* = 5, g *N* = 9, h *N* = 12, i *N* = 8, j *N* = 4, k *N* = 13, l *N* = 15

The pharmacokinetics of 50 mg alisertib administered alone were similar in both treatment arms of Study 1 with median T_max_ of 3 to 4 h, and mean t_1/2_ of 16 h. Exposure parameters (C_max_ and AUC parameters) for alisertib were also similar between the arms. Variability of alisertib pharmacokinetics was moderate with CV% of 23% to 43% for C_max_ and AUC parameters. Metabolite M1 had similar median T_max_ (4 h) and mean t_1/2_ (approximately 15 to 18 h) to alisertib and exposure was approximately 22% to 29% of alisertib exposure (based on comparison of mean AUC_0-last_ in each arm). Variability for metabolite M1 was generally higher than for alisertib with CV% values for C_max_ and AUC parameters of 74% to 164%. Median T_max_ of metabolite M2 was 10 h (ie, later than the parent). It also appeared to have a longer t_1/2_ (mean values of 19.1 to 22 h) although it should be noted that the number of patients with evaluable data was low (*N* = 4 or 5) in both treatment arms and hence these values should be interpreted with caution. Metabolite M2 exposure was approximately 31 to 45% of alisertib exposure (based on comparison of AUC_0-last_ in each arm). Variability for metabolite M2 was generally higher than for alisertib with CV% values for C_max_ and AUC_0-last_ of 37% to 65%. CV% for metabolite M2 was lower for AUC_0-inf_ but this is likely due to the low number of patients with evaluable data for this parameter.

As expected, alisertib exposure in Study 2 was lower than in Study 1 due to the lower dose (30 mg cf. 50 mg). Median T_max_ was approximately 3 h and mean t_1/2_ was approximately 23 h. Variability of C_max_ and AUC parameters was similar across the 2 studies for alisertib and both of its major metabolites.

Following a single oral dose of 50 mg alisertib in Study 1, the median T_max_ of alisertib was 3 h in the presence of esomeprazole compared to 4 h when administered alone, and the mean t_1/2_ was similar (approximately 16 h) in the presence and absence of esomeprazole. Statistical comparison of alisertib exposure parameters suggested that esomeprazole increased the AUCinf by 28% (Table 1). The geometric mean ratio for alisertib C_max_ was 1.14 suggesting a small increase in the presence of esomeprazole, although the 90% CIs included 1.00.

Following a single oral dose of 50 mg alisertib in Study 1, the median T_max_ of alisertib was approximately 2 h in the presence of rifampin compared to 4 h when administered alone, and the mean t_1/2_ was approximately 8 h compared to approximately 16 h when administered alone. Statistical comparison of exposure parameters suggested that rifampin did not alter alisertib C_max_ but decreased AUCinf by approximately 50% (Table 1). Although not statistically compared, the presence of rifampin did appear to increase exposure of metabolite M1 (approximately 2.8-fold increase in mean C_max_, and 1.6-fold increase in mean AUC_0-last_). Mean t_1/2_ of metabolite M1 was 12 h in the presence of rifampin compared to 18 h in the absence of rifampin. The mean AUC_0-last_ for metabolite M2 did not increase in the presence of rifampin although mean C_max_ did increase (by approximately 1.8-fold). Mean AUC_0-inf_ was increased and mean t_1/2_ for M2 was decreased in the presence of rifampin but again the number of subjects with evaluable data in the absence of rifampin was low, making interpretation of these data difficult.

Following a single oral dose of 30 mg alisertib in Study 2, the median T_max_ of alisertib was 2.9 h in the presence and absence of itraconazole. Alisertib mean t_1/2_ was approximately 25 h in the presence of itraconazole compared to approximately 23 h when administered alone. Statistical comparison of alisertib exposure parameters suggested that itraconazole did not alter alisertib C_max_, but increased the AUC parameters by approximately 35 to 40%, although the CIs were wide with the lower bound being around 1.00 (Table 1). Although not statistically compared, the presence of itraconazole did not alter the mean C_max_ of metabolite M1, but did appear to increase the mean AUC parameters (approximately 1.4- and 1.2-fold increases in mean AUC_0-last_, and AUC_0-inf_, respectively). The mean C_max_ and AUC parameters for metabolite M2 were slightly lower (0.8- to 0.9-fold) in the presence of itraconazole compared to when alisertib was administered alone.

### Safety

In Study 1, a total of 52 (95%) patients had at least 1 adverse event and 43 (78%) patients had treatment-related adverse events (note that for both studies, ‘treatment’ included both alisertib and the interactants). The most common treatment-related adverse events (>30% overall) were diarrhea, alopecia, and neutropenia (Table 2). Most of the adverse events were more common in the esomeprazole treatment arm than the rifampin arm. Four patients died during the study; none of the deaths were deemed related to study treatment and all were attributed to progression of patient’s cancers or complications due to progressive disease. Five patients had other serious adverse events that were considered to be treatment related; 2 patients in the esomeprazole arm and 3 patients in the rifampin arm. The most common of these other treatment-related serious adverse events were nausea and vomiting which were both reported by 2 patients.

**Table 2:** Most commonly reported (≥10% total) treatment-related adverse events (Study 1)

Adverse Events (Preferred Terms)	Esomeprazole + Alisertib *N* = 26 n (%)	Rifampin + Alisertib *N* = 29 n (%)	Total *N* = 55 n (%)
Patients with at least 1 drug-related treatment-emergent adverse event	25 (96)	18 (62)	43 (78)
Diarrhea	14 (54)	4 (14)	18 (33)
Alopecia	11 (42)	6 (21)	17 (31)
Neutropenia	11 (42)	6 (21)	17 (31)
Fatigue	8 (31)	6 (21)	14 (25)
Stomatitis	10 (38)	4 (14)	14 (25)
Anaemia	7 (27)	3 (10)	10 (18)
Nausea	5 (19)	5 (17)	10 (18)
Decreased appetite	6 (23)	2 (7)	8 (15)
Vomiting	5 (19)	3 (10)	8 (15)
Leukopenia	3 (12)	4 (14)	7 (13)
Thrombocytopenia	4 (15)	3 (10)	7 (13)
Dehydration	5 (19)	1 (3)	6 (11)

All patients received single and multiple doses (7 days) of alisertib in 2 treatment cycles. Patients also received either esomeprazole or rifampin in cycle 2. Drug-related includes events potentially related to alisertib, esomeprazole and/or rifampin

Adverse events were coded using the Medical Dictionary for Regulatory Activities (MedDRA) version 17.0

In Study 2, a total of 23 (96%) patients had at least 1 adverse event and 16 (67%) patients had treatment-related adverse events. The most common treatment-related adverse event (>30%) was nausea (Table 3). One patient died during the study from glioblastoma which was not considered to be treatment-related. Drug-related serious adverse events (SAEs) occurred in 3 (13%) patients including vomiting, melena, disseminated herpes zoster, and sepsis, which occurred in 1 (4%) patient each.

**Table 3:** Most commonly reported (≥10%) treatment-related adverse events (Study 2)

Adverse Events (Preferred Terms)	30 mg Alisertib + Itraconazole (Part A) 50 mg BID Alisertib (Part B) *N* = 24 n (%)
Patients with at least 1 drug-related treatment-emergent adverse event	16 (67)
Nausea	8 (33)
Diarrhoea	6 (25)
Fatigue	5 (21)
Vomiting	5 (21)
Alopecia	4 (17)
Neutropenia	4 (17)
Stomatitis	4 (17)
Anaemia	3 (13)
Constipation	3 (13)

Adverse events were coded using the Medical Dictionary for Regulatory Activities (MedDRA) version 18.0

In both studies, decreases were observed in leukocytes and neutrophils over the course of the study which is consistent with the known safety profile of alisertib (Takeda data on file). There were no notable trends observed for vital signs or ECG findings. As expected, the ECOG status showed changes over the studies, which in most cases reflected a worsening condition with a difference from screening by 1 score.

## Discussion

Polypharmacy is common in cancer patients with advanced disease. Concomitant medications that are likely to produce clinically significant alterations in anticancer agent exposure can have an adverse impact on their safety and efficacy. In the case of cytotoxic agents with narrow therapeutic windows, clinical DDIs can be potentially life threatening. Therefore, clinical development of anticancer drugs requires unique considerations for management of DDI risks as well as for the conduct of controlled pharmacokinetic DDI studies to guide risk management in later-phase clinical trials and eventually inform product labelling [16]. Herein, we present the results of 3 clinical DDI evaluations with alisertib as the potential victim drug that were designed based on understanding of factors that may alter alisertib exposure through modulation of its absorption or clearance.

Alisertib is an acidic drug with pH-dependent solubility and is formulated as an enteric coated tablet. Accordingly, one of the DDI evaluations was designed to evaluate the effects of the PPI esomeprazole on alisertib PK, in order to assess the risk for potential DDIs with gastric acid reducing agents. Esomeprazole produced a 28% increase in alisertib systemic exposure. The mean t_1/2_ of alisertib following a single dose with or without esomeprazole was similar, indicating that esomeprazole did not alter alisertib systemic clearance, consistent with the interaction being at the level of alisertib absorption. The observed increase in alisertib exposure upon administration of a gastric acid-reducing agent is consistent with alisertib being an acidic drug with potential for increased solubility under less acidic conditions.

These results support the recommendation that gastric acid-reducing agents be avoided in patients receiving alisertib, given that the recommended dose of alisertib for clinical development is its MTD, suggesting a limited therapeutic window. Specifically, clinical studies of alisertib recommend avoiding use of PPIs as their effect on intra-gastric pH is long lasting, secondary to covalent binding to the gastric [H^+^/K^+^] ATPase. However, patients in alisertib clinical studies requiring intermittent use of gastric acid reducing agents are permitted to use H_2_-receptor antagonists (e.g., famotidine, ranitidine) on non-dosing days (ie, during treatment-free rest periods when alisertib is not administered) due to the reversible mode of action of these agents and shorter duration of gastric acid suppression. On alisertib dosing days, patients are allowed to take neutralizing antacids with the exception of a 4-h window around alisertib dosing (from 2 h before until 2 h after dosing) when the use of such antacids is to be avoided. These protocol-specified risk management guidelines are designed based on understanding of the differential pharmacology and duration of effect on gastric pH among various modalities used to manage dyspepsia and gastric acid reflux.

Administration of the strong metabolic enzyme inducer rifampin (600 mg daily for 10 days) to patients with cancer decreased single-dose plasma alisertib exposure (AUC) by approximately 50%. The mean t_1/2_ of alisertib following a single dose is 8 h and 16 h in the presence and in the absence of rifampin, respectively. Rifampin did not alter the peak concentration of alisertib. The reduction in total systemic exposure of alisertib and shortening of half-life observed upon co-administration with rifampin indicates the contribution of PXR-inducible enzymes to the clearance of alisertib in humans. This is consistent with the observation of both oxidative and conjugative metabolites of alisertib in a mass balance study (Takeda data on file), the documentation of circulating exposures of both the primary glucuronide metabolite M1 and the primary oxidative metabolite M2 in this study, and the knowledge of rifampin being a pleiotropic inducer of multiple drug-metabolizing CYP and UGT enzymes. The metabolite/parent ratios for both M1 and M2 were increased following rifampin administration, consistent with induction of both the oxidative and conjugative metabolic pathways of alisertib. These results support the conclusion that chronic use of concomitant strong inducers of PXR-inducible enzymes be avoided in patients receiving alisertib.

The strong CYP3A inhibitor itraconazole produced an approximately 40% increase in total alisertib systemic exposure. These results are consistent with a partial contribution of CYP3A-mediated metabolism to the overall clearance of alisertib in humans. The observed increase in AUC without corresponding increase in C_max_ further indicates that the interaction between itraconazole and alisertib is likely explained mainly via inhibition of systemic clearance as opposed to decreased presystemic extraction by the intestine and/or liver. These observations are consistent with alisertib being a low clearance compound (apparent oral clearance of 4.25 L/h [3] and also indicate that while hepatic CYP3A is a partial contributor to alisertib clearance, intestinal CYP3A is unlikely to contribute meaningfully to the presystemic extraction of orally administered alisertib. The systemic exposures of M2/parent ratio were decreased following multiple dose administration of itraconazole, consistent with inhibition of oxidative metabolism of alisertib.

The 40% increase in total systemic exposure of alisertib can be inferred to be of clinical relevance, as alisertib is a cytotoxic agent administered at its maximum tolerated dose in clinical development. Accordingly, the standard starting dose of alisertib is not recommended in patients requiring coadministration of strong CYP3A inhibitors, and an appropriately reduced starting dose may need to be considered if continued treatment with strong CYP3A inhibitors cannot be avoided. Specifically, the observed 40% increase in AUC in the presence of itraconazole suggests that a 30% reduction in alisertib starting dose (i.e., 35 mg BID instead of 50 mg BID standard dose in the single agent setting) in patients requiring treatment with strong CYP3A inhibitors can be expected to normalize systemic exposures to the general patient population.

Overall, the results of the DDI studies presented here collectively provide a holistic characterization of the effects of extrinsic factors on alisertib PK. These findings have been crucial in informing risk management for DDIs with gastric acid reducing agents, CYP3A inhibitors and inducers in the alisertib clinical development program.

## References

[1] Dees EC, Cohen RB, von Mehren M, Stinkchcombe TE, Liu H, Venkatakrishnan K et al (2012) Phase 1 study to aurora a kinase inhibitor MLN8237 in advance solid tumors: safety, pharmacokinetics, pharmacodynamics, and bioavailability of two oral formulations. Clin Cancer Res 18:4775–4784. doi:10.1158/1078-0432.CCR-12-0589 10.1158/1078-0432.CCR-12-0589 22767670

[2] Cervantes A, Elez E, Roda D, Ecsedy J, Macarulla T, Venkatakrishnan K, Roselló S et al (2012) Phase I pharmacokinetic/pharmacodynamic study of MLN8237, an investigational, oral, selective aurora a kinase inhibitor, in patients with advanced solid tumors. Clin Cancer Res 18:4764–4774. doi:10.1158/1078-0432.CCR-12-0571 10.1158/1078-0432.CCR-12-0571 22753585

[3] Venkatakrishnan K, Zhou X, Ecsedy J, Mould DR, Liu H, Danaee H et al (2015) Dose selection for the investigational anticancer agent alisertib (MLN8237): pharmacokinetics, pharmacodynamics, and exposure-safety relationships. J Clin Pharmacol 55:336–347. doi:10.1002/jcph.410 10.1002/jcph.410 25302940

[4] Friedberg JW, Mahadevan D, Cebula E, Persky D, Lossos I, Agarwal AB et al (2014) Phase II study of alisertib, a selective aurora a kinase inhibitor, in relapsed and refractory aggressive B- and T-cell non-Hodgkin lymphomas. J Clin Oncol 32:44–50. doi:10.1200/JCO.2012.46.8793 10.1200/JCO.2012.46.8793 PMC3867644 24043741

[5] Goldberg SL, Fenaus P, Craig MD, Gyan E, Lister J, Kassis J et al (2014) An exploratory phase 2 study of investigational aurora a kinase inhibitor alisertib (MLN8237) in acute myelogenous leukemia and myelodysplastic syndromes. Leuk Res Rep 3:58–61. doi:10.1016/j.lrr.2014.06.003 10.1016/j.lrr.2014.06.003 PMC4110881 25068104

[6] Matulonis UA, Sharma S, Ghamande S, Gordon MS, Del Prete SA, Ray-Coquard I et al (2012) Phase II study of MLN8237 (alisertib), an investigational aurora a kinase inhibitor, in patients with platinum-resistant or -refractory epithelial ovarian, fallopian tube, or primary peritoneal carcinoma. Gynecol Oncol 127:63–69. doi:10.1016/j.ygyno.2012.06.040 10.1016/j.ygyno.2012.06.040 22772063

[7] Melichar B, Adenis A, Lockhart AC, Bennouna J, Dees EC, Kayaleh O et al (2015) Safety and activity of alisertib, an investigational aurora kinase a inhibitor, in patients with breast cancer, small-cell lung cancer, non-small-cell lung cancer, head and neck squamous-cell carcinoma, and gastro-oesophageal adenocarcinoma: a five-arm phase 2 study. Lancet Oncol 16:395–405. doi:10.1016/S1470-2045(15)70051-3 10.1016/S1470-2045(15)70051-3 25728526

[8] Owonikoko TK, Nackaerts K, Csoszi T, Ostoros G, Baik C, Mark Z et al (2016) Randomized phase 2 study of investigational aurora a kinase (AAK) inhibitor alisertib (MLN8237) + paclitaxel (P) vs placebo + P as second line therapy for small-cell lung cancer (SCLC). Ann Oncol 27:14230. doi:10.1093/annonc/mdw389.01

[9] Nui H, Manfredi M, Ecsedy JA (2015) Scientific rationale supporting the clinical development strategy for the investigational aurora a kinase inhibitor alisertib in cancer. Front Oncol 5:1–9. doi:10.3389/fonc.2015.00189 10.3389/fonc.2015.00189 PMC4547019 26380220

[10] Zhou X, Pusalkar S, Chowbhury S, Searle S, Li Y Mertz J et al (2014) Mass balance, routes of excretion and pharmacokinetics of investigational oral [14C]alisertib (MLN8237) in patients with advanced solid tumors or lymphoma. Mol cancer Ther 12(11_Supplement):B216. doi: 10.1158/1535-7163.TARG-13-B216

[11] US Food and Drug Administration. Guidance for Industry (2012) Drug Interaction Studies – Study design, data analysis, implications for dosing, and labelling recommendations http://www.fda.gov/downloads/Drugs/GuidanceComplianceRegulatoryInformation/Guidances/UCM292362.pdf. Accessed 20 Dec 2016

[12] European Medicines Agency. Guideline on the Investigation of drug interactions. Final 2012 CPMP/EWP/560/95/Rev. 1 Corr.* http://www.ema.europa.eu/docs/en_GB/document_library/Scientific_guideline/2012/07/WC500129606.pdf. Accessed 20 Dec 2016

[13] Sachs G, Shin JM, Howden CW (2006) Review article: the clinical pharmacology of proton pump inhibitors. Aliment Pharmacol Ther 23(s2):2–8 10.1111/j.1365-2036.2006.02943.x 16700898

[14] Smelick GS, Heffron TP, Chu L, Dean B, West DA, DuVall SL et al (2013) Prevalence of acid-reducing agents (ARA) in cancer populations and ARA drug–drug interaction potential for molecular targeted agents in clinical development. Mol Pharm 10:4055–4062. doi:10.1021/mp400403s 10.1021/mp400403s 24044612

[15] Budha NR, Frymoyer A, Smelick GS, Jin JY, Yago MR, Dresser MJ et al (2012) Drug absorption interactions between oral targeted anticancer agents and PPIs: is pH-dependent solubility the Achilles heel of targeted therapy? Clin Pharmacol Ther 92:203–213. doi:10.1038/clpt.2012.73 10.1038/clpt.2012.73 22739140

[16] Venkatakrishnan K, Pickard MD, von Moltke LL (2010) A quantitative framework and strategies for management and evaluation of metabolic drug-drug interactions in oncology drug development: new molecular entities as object drugs. Clin Pharm 49:703–727. doi:10.2165/11536740-000000000-00000 10.2165/11536740-000000000-00000 20923246

[17] Liu L, Bello A, Dresser MJ, Heald D, Komjathy SF, O'Mara E et al (2016) Best practices for the use of itraconazole as a replacement for ketoconazole in drug-drug interaction studies. J Clin Pharmacol 56:143–151. doi:10.1002/jcph.562 10.1002/jcph.562 26044116

